# Numerical Technique for Study of Noise Grating Dynamics in Holographic Photopolymers

**DOI:** 10.3390/polym12112744

**Published:** 2020-11-19

**Authors:** Robert R. McLeod

**Affiliations:** Electrical Computer and Energy Engineering Department and Materials Science and Engineering Program, UCB 425, University of Colorado Boulder, Boulder, CO 80309, USA; mcleod@colorado.edu

**Keywords:** holography, photopolymer, scatter 3, signal-to-noise, haze, noise gratings

## Abstract

Although the angular distribution of noise gratings in holographic photopolymer is understood to arise from Bragg matching, the details of scatter strength and dynamics are not fully understood. This confounds development of materials and recording techniques that minimize haze. Here, the kinetics are studied using a multi-physics numerical approach coupling diffraction of light from the dynamic material including scatter centers, reactions of chemical species initiated by this light, diffusion and swelling of these constituents, and the formation of the refractive index from the resulting composition. The approach is validated in the case of two-beam transmission holography by comparison to traditional harmonic series and rigorous coupled-mode approaches. Two beam holography in the presence of scatter is then used to study haze development. This reveals that haze due to weak noise gratings grows significantly above initial scatter only in reaction-limited materials, consistent with proposed Bragg-matched amplification mechanisms. Amplified haze is found to be proportional to initial scatter, quantifying the impact of clean sample fabrication. Conversely, haze is found to grow super-linearly with sample thickness, illustrating the significant challenge for applications requiring low haze in large thickness.

## 1. Introduction

Most applications of holographic photopolymers require high diffraction efficiency but also low scatter. Examples include data storage, where scatter contributes to the bit error rate, and augmented reality where scatter contributes to haze that detracts from visibility. Unfortunately, it is commonly observed in material development that these two quantities are coupled. That is, high dynamic range materials capable of recording high diffraction efficiency also typically develop large scatter. It is thus a significant challenge to develop materials formulations and processing methods resulting in a high signal to noise ratio (SNR).

Previous theoretical studies of scatter development in holographic photopolymers have identified noise gratings seeded by initial scatter centers as the source of recorded haze [1,2]. The central concept of these studies is that recorded scatter must be considered as an ensemble of weak holograms, a small fraction of which are Bragg-selectively replayed by the reference and object beams. This amplifies a fraction of the scatter which then interferes with the writing beams to write further noise gratings. If grating development is fast in comparison to the recording rate, this process dynamically forms characteristic arcs and lines of scatter at distinct angles. In contrast, an approach where particles are thought of as seeds for self-written spatial filaments [3] would be expected to scatter approximately isotopically. Reported experimental scatter patterns are generally consistent with the former theory, showing strong angular dependence in narrow arcs and lines consistent with Bragg-degenerate noise grating growth. Monte Carlo numerical studies are consistent with these results [4].

**Figure 1:**
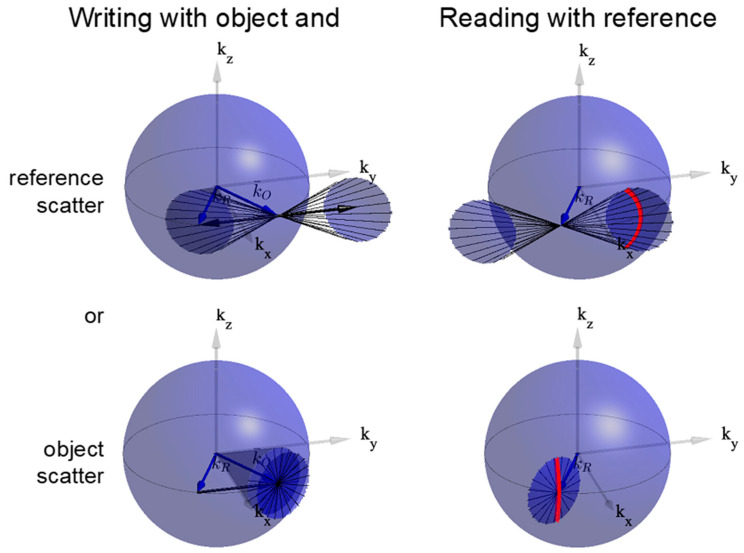
Accepted process for writing (**left**) and Bragg-matched readout (**right**) of noise gratings, illustrated in momentum space for two-beam holography. Scattered fields from the reference (**top**) or object (**bottom**) beam, shown as shaded cones, interfere with the object to write primary and complex-conjugate grating manifolds, shown as thin black lines. When these noise gratings are read out by the reference beam (right), the diffraction is Bragg-matched at the intersection of the k-sphere and the spherical manifolds, marked in red. Observed scatter patterns [1,2,7] are consistent with this process.

The observed scatter patterns can be explained by the Ewald sphere analysis shown in Figure 1. In the left two images, object and reference beams, shown as blue arrows, write a holographic grating with positive and negative side bands, shown as the two heavy black arrows. Scatter in a cone around the reference beam (top) or object beam (bottom), shown as a circular patch on the Ewald sphere, interferes with the object beam to write weak noise gratings. The positive and negative side bands of these noise gratings, shown as thin black lines, reside on two-dimensional spherical surfaces centered on the holographic grating K vector. The interference of the object beam with the reference (top) and object (bottom) scatter thus generates four two-dimensional noise grating manifolds, shown as shaded spherical patches. The right-hand plots show that a curved one-dimensional slice of two of these manifolds is Bragg matched when replayed by the reference beam. The slice, shown as heavy red lines, is found at the intersection of the Ewald sphere and the diffracted k vectors that occupy the curved spherical manifolds. Within these arcs, Bragg-matched diffraction of the reference beam off of the noise grating manifolds increases the magnitude of the noise. This larger scattered intensity writes new and stronger noise holograms and the process repeats, amplifying the scatter in a distinct pattern. The figure shows only half of the possible noise grating manifolds for clarity. Interreference of the reference beam with scatter follows the same process, mirrored around the *xz* plane.

While this analysis and the previous studies explain the angular distribution of recorded scatter patterns, they do little to guide materials and process development that would maximize SNR. The amplitude of the noise gratings is expected to be strongly dependent on the statics of the scatter centers, the dynamics of the recording process, Bragg selectivity of the noise gratings, and recording parameters such as period and intensity. These are difficult to study experimentally since it is challenging to know or precisely control the details of the initial weak scattering. Additionally, experimentally exploring the parameter space of materials formulations and processing conditions is costly when it is not clear what variables should be probed and in what ranges they would be expected to impact the SNR.

The goal of this paper is to therefore to establish a numerical approach to study these questions. Because the observed Bragg-matched noise gratings have structure in both transverse dimensions and the amplification process depends on growth in both depth and time, such a study requires a simulation in three spatial dimensions and time. Conversely, numerical studies of predominantly local effects such as phase segregation in holographic polymers have typically been restricted to a slice through the hologram in the plane of the grating wave vector and time [5].

For generality, we will simulate a specific model material broadly similar to widely available commercial formulations [6]. The goal is to capture the minimal materials and processing physics in order to establish scaling relationships and elucidate possibly fruitful approaches to experimental reduction of haze. In the following sections, we illustrate typical haze dynamics and angular dependence in a model material. We then describe a multi-physics code suite created to simulate signal and noise growth in this material. The code is validated by comparing to a closed-form solution to two-beam grating growth via a harmonic expansion solution to the chemical reaction/diffusion equations. Finally, we explore noise dynamics with this tool to make predictions about noise growth versus experimentally accessible parameters.

## 2. Methods

To study the how coherent light illuminating a scattering, photosensitive medium drives the development of both signal and noise, we implement a multi-physics code suite. The technique models the holographic recording process by iterating four primary calculations as a function of exposure time. These are (1) the Fourier beam-propagation (FBPM) method used to calculate optical propagation and diffraction within the 3D index structure, (2) a discrete time-stepping solution of the first-order photochemical rate equations, (3) diffusion of mobile species via a fast-Fourier transform (FFT) solution of Fick’s law and (4) update of the material index via an a priori model for two-component holographic photopolymers. We describe each step in more detail below.

### 2.1. Sample Preparation Including Scatter Centers

**Figure 2:**
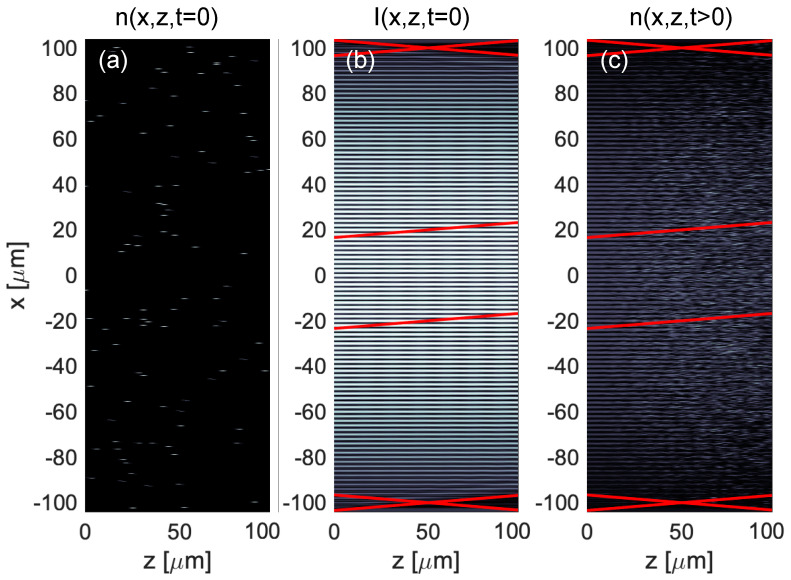
Slice at y = 0 through the three-dimensional numerical space illustrating the numerical method employed. An initial index distribution of randomly distributed scatter centers (**a**) is created to seed the formation of noise gratings. This is illuminated from the left with two tilted coherent Gaussian beams that interfere as they propagate in thickness, z, forming the intensity pattern (**b**). The downward sloped red lines (top and bottom) indicate the 1/e waist of the object beam electric field while the upward tilted red lines (top and bottom) indicate the 1/e waist of the reference beam electric field. The upward tilted red lines (center) indicate the 1/e waist of the probe field that will be used to read out the eventual hologram. The interference of object and reference fields drives the photochemical reaction, diffusion and index-formation process, resulting in an index distribution (**c**) consisting of the hologram, haze and the static scatter centers. The visible evolution of the fringes from low to high noise as a function of depth illustrates the interplay between these structures within the material.

The substrates and recording material are assumed to contain photo-insensitive scattering centers that initiate noise gratings. These may be scratches in the substrate, dust, or incompletely mixed components of the formulation, for example. These scatter centers can be described either by shape (e.g., microspheres) or via their angular scatter distribution (e.g., Rayleigh). In the latter case, the microscopic shape of the scatter center sampled on the computational grid is found by inverse Fourier transforming the specified angular scatter distribution. Identical copies of these shapes are randomly distributed throughout the 3D recording medium and/or substrates with a specified number density. Given the shape and number density, the cross-section of the scatter centers is adjusted to create a specified initial haze. Figure 2a shows a representative cross section of the polymer refractive index illustrating the randomly distributed scattering particles before any exposure.

### 2.2. Holographic Exposure

For each time step of the exposure, two coherent Gaussian writing beams, symmetrically tilted relative to the surface normal, are propagated through the depth of the material using the non-paraxial FBPM method. The x and y spatial dimensions are at least Nyquist sampled relative to the optical wavelength in the material in order to capture scattering angles over the full forward hemisphere. The writing beam angle is chosen to yield a transmission hologram with period, Λ, sufficiently larger than the grid size such that individual grating fringes are adequately sampled. The writing beams attenuate due to initiator absorption, coherently interfere, scatter off of both the static microparticles and dynamically recorded noise gratings, and diffract off of the developing hologram. The three-dimensional optical intensity at each time step is calculated from the magnitude squared of the electric field, as shown in Figure 2b.

### 2.3. Photochemical Reactions

This intensity then drives a sequence of chemical reactions, calculated through time-stepping the first order chemical rate equations. The first reaction in the sequence is the photolysis of an initiator (assumed to be present in sufficient excess that it does not significantly deplete) by the optical intensity to generate primary radicals with rate constant $k_{I}$, which rapidly react to form a radical monomer $m^{*}$. These radicals diffusive with diffusivity *D* and terminate via a unimolecular reaction with rate constant $k_{T}$. We chose a unimolecular termination reaction to match the intensity reciprocity observed for two-component holographic photopolymers [8]. To our knowledge, the linear and reciprocal intensity response of these materials has not been adequately explained in the literature and is surprising given the otherwise universal sublinear intensity response of radically initiated photopolymers. With these assumptions, the radical concentration evolves as:

(1) $$\frac{d}{dt}\left[m^{*}\left(\vec{r},t\right)\right]=D\nabla^{2}\left[m^{*}\left(\vec{r},t\right)\right]-k_{T}\left[m^{*}\left(\vec{r},t\right)\right]+k_{I}I\left(\vec{r},t\right).$$

**Figure 3:**
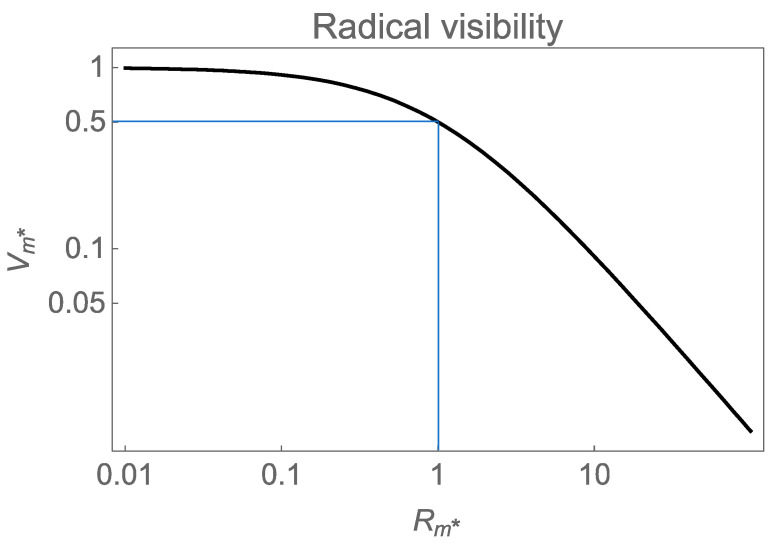
Radical fringe visibility, $V_{m^{*}}$, as a function of diffusion rate normalized to termination rate, $R_{m^{*}}$. A significant concentration of radicals is found in the dark when the diffusion rate across a period is larger than the termination rate. The fringe visibility and, thus, the holographic index is expected to fall like $\Lambda^{-2}$ for a sufficiently small period Λ.

The termination rate constant $k_{T}$ is assumed to encompass any first order process which prevents radicals from participating in the subsequent propagation reaction. In steady state, the solution to this equation under sinusoidal illumination, $I\left(x\right)=\bar{I}\;\left[1+\cos\left(k\;x\right)\right]$, is found to be a sinusoid with finite visibility:

(2) $$\left[m^{*}\left(\vec{r},t\right)\right]=\frac{k_{I}\;}{k_{T}}\bar{I}\;[1+V_{m^{*}}\cos\left(k\;x\right)],$$

where $\bar{I}$ is the average intensity, k≡2π/Λ is the grating wave number and the radical visibility is given by:

(3) $$V_{m^{*}}\equiv \frac{1}{R_{m^{*}}+1}.$$

where $R_{m^{*}}\equiv Dk^{2}/k_{T}$ is the diffusion rate of radicals across a period relative to radical termination rate. As shown in Figure 3, the distribution of radicals is identical to the sinusoidal intensity ($V_{m^{*}}=1$) when this unitless ratio is much smaller than 1. However, when diffusion is sufficiently fast that radicals can diffuse into the dark region of the fringe before terminating $\left(R_{m^{*}}\gg 1\right)$, the visibility of the radical distribution decreases inversely proportional to period squared. This behavior has previously been identified as the resolution limit in published materials [9]. This explains the observation that significant improvement in dynamic range can be achieved by increasing the termination rate via radical traps on the matrix [10]. Here we will assume the steady-state distribution of radicals with finite visibility given by Equation (3), governed by the unitless diffusion rate to termination rate ratio $R_{m^{*}}$.

These radicals then drive the propagation reaction with rate constant $k_{P}$, depleting the mobile monomer as:

(4) $$\frac{d}{dt}\left[m\left(\vec{r},t\right)\right]=D\nabla^{2}\left[m\left(\vec{r},t\right)\right]-k_{P}\left[m\left(\vec{r},t\right)\right]\left[m^{*}\left(\vec{r},t\right)\right].$$

The concentration of polymer $\left[P\left(\vec{r},t\right)\right]$ is found by the time integral of the final term in the Equation (4). This assumes that all polymer is immobilized, e.g., by hydrogen bonding, entanglement or an explicit reactive group on the matrix. Oligomeric species are not tracked, simplifying the description of the propagation reaction to a single rate equation. Note that polymer concentration is defined to be the concentration of monomer that has reacted such that, neglecting diffusion, the sum of monomer and polymer is conserved as $\left[m\right]+\left[P\right]=\left[m_{0}\right]$, where $\left[m_{0}\right]$ is the formulated monomer concentration. Extensions of this approach to more fully describe the polymer development have been extensively studied [11]. The goal here is to capture the essential chemical physics of the material with the minimal description that will reveal scaling relations governing the dynamics of recording noise. Details of the time-stepping scheme for Equations (1) and (4) are given in Appendix A.

### 2.4. Monomer Diffusion

Mobile monomer must diffuse to implement the first term in Equation (4). The three-dimensional distribution of monomer is Fourier transformed via the FFT and multiplied by the transfer function of Fick’s second law in homogenous space, then inverse transformed. Assuming that diffusivity, *D*, is independent of space and time, that transfer function is $H\left(f_{x},f_{y},f_{z}\right)=\exp\left[-\left(f_{x}^{2}+f_{y}^{2}+f_{z}^{2}\right)/f_{c}^{2}\right]$ where $f_{x,y,z}$ are the Cartesian spatial frequencies and the characteristic spatial frequency of diffusion in a single time step, δt, is given by $f_{c}=1/(2\;\pi \sqrt{D\;\delta t})$. Note that H(0,0,0)=1 enforces the conservation of mass.

### 2.5. Index Development

The index of the photopolymer is then calculated from these chemical distributions. The local volume fraction of polymer is first found from the fractional conversion and the known polymerization shrinkage. The volume fraction of the rubbery solid matrix is found assuming perfect mixing and incompressibility as one minus the monomer and polymer volume fractions. Finally, the index of the three-component mixture is calculated by the Lorentz–Lorenz equation:

(5) $$\frac{n^{2}-1}{n^{2}+2}=\underset{i}{\sum }\frac{n_{i}^{2}-1}{n_{i}^{2}+2}\phi_{i},$$

where the refractive indices $n_{m},\;n_{P},\;n_{M}$ of the monomer, polymer and matrix, respectively, are found using prism-coupler measurements of mixtures of the components. These are typically not the same as measurements of pure compounds. For example, vitrification typically limits complete shrinkage upon polymerization. Conversely, in the rubbery host, the writing monomer can typically fully convert with minimal shrinkage stress. This yields a greater shrinkage and refractive index than exhibited by neat polymer at full conversion. Taking the fractional volume shrinkage upon polymerization to be σ, the associated volume fractions of each component are:

(6) $$\phi_{m}=\phi_{m0}m\left(\vec{r},t\right),\;\phi_{P}=\phi_{m0}\left(1-\sigma \right)P\left(\vec{r},t\right),\;\phi_{M}=1-\phi_{m0}\left[m\left(\vec{r},t\right)+\left(1-\sigma \right)P\left(\vec{r},t\right)\right],$$

where $\phi_{m0}$ is the monomer volume fraction before exposure and $m=\left[m\right]/\left[m_{0}\right]$ and $P=\left[P\right]/\left[m_{0}\right]$ are the concentration of monomer and polymer normalized to the initial monomer concentration. A cross-section of the typical resulting index is shown in Figure 2c.

An important aspect of this calculation is that index change and shrinkage upon polymerization result in finite index contrast immediately upon polymerization. Subsequent diffusion amplifies this response. Since the development of haze is hypothesized to be self-reinforced noise gratings that form during exposure, the time dynamics of the index formation process are critical.

### 2.6. Holographic and Haze Readout

**Figure 4:**
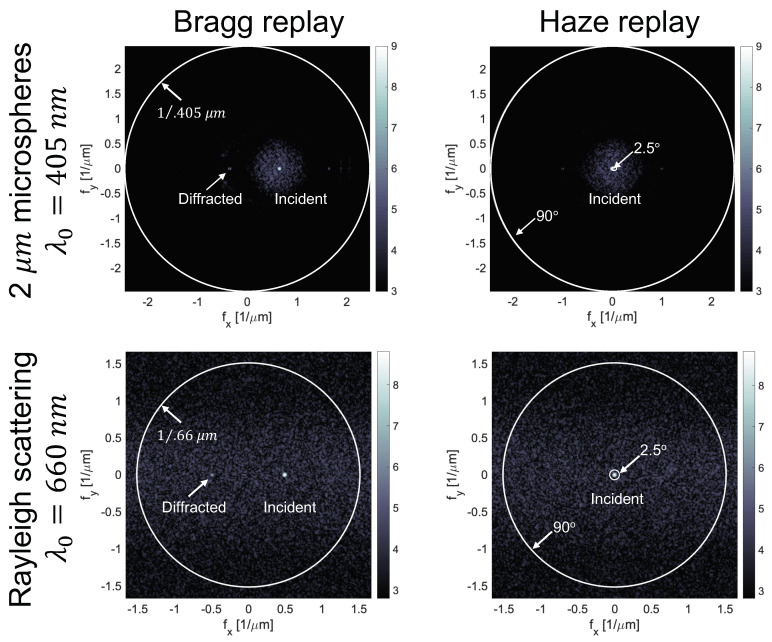
Intensity of holographic readout (**left**) and haze readout (**right**) for a transmission grating with 1 µm period written at 405 nm with both beams linearly polarized along the y direction. The material scatters due to embedded 2 μm diameter microspheres (**top**) or Rayleigh scattering (**bottom**). To illustrate the impact of reading wavelength, the top and bottom images use 405 and 660 nm, respectively. The diffracted light exiting the sample in both cases is rendered in the Fourier domain just inside the exit face of the material. The spatial frequencies corresponding to the angular limits of haze integration in air are indicated by circles. For the microspheres, scatter is observed in a small scatter cone around each beam. Conversely, Rayleigh scattering is uniform in the s or $f_{x}$ plane and has cos(θ) distribution in the *p* or $f_{y}$ plane.

Finally, the efficiency of the hologram is found by illuminating the material index. This index is taken to be the sum of the static (scattering) and dynamic (holographic photopolymer) index distributions. A Bragg matched reference beam incident on the surface z = 0 is propagated through the volume with FBPM. The diffracted intensity is integrated at the exit face in an angular range centered on the object beam. The readout reference beam at 405 or 660 nm is assumed to be sufficiently weak and/or outside the initiator absorption spectrum that it does not modify the index distribution. Next, a normally incident beam is used to measure the haze. Haze is calculated, per ASTM standard D1003, as the fraction of the power scattered by the sample out of a normally incident probe for all angles (measured from the normal) greater than 2.5° and less than 90°. Typical angular spectra of the intensity exiting the material in both cases are illustrated in Figure 4.

### 2.7. Post Exposure Development

Finally, after the exposure is complete, monomer is allowed to diffuse, becoming spatially uniform. This step simulates dark development time and assumes no limits to monomer transport due to finite solubility in high conversion regions. The uniform monomer is then fully converted to polymer, simulating a perfectly incoherent and spatially uniform flood cure. Quantitative conversion is assumed, that is no monomer remains. A Bragg selectivity curve is then calculated by measuring the diffraction efficiency as a function of reference beam angle.

## 3. Validation

To ensure the appropriateness and proper implementation of this combination of methods, it is critical that the code suite be validated. There are limited theoretical methods for predicting scatter development in these materials and the problem is inherently statistical. Thus, we instead validate the technique using a fully theoretical model of two-beam Bragg holography. A comparison to theory is chosen rather than experiment to enable direct comparison with no fitting to find parameters that are not always easily accessible experimentally, e.g., diffusion and reaction rates.

Combining Equations (2) and (4) results in a single rate equation governing this simplified system. Restricting the analysis to one dimension in the plane of incidence gives:

(7) $$\frac{d}{dt}\left[m\left(x,t\right)\right]=D\nabla^{2}\left[m\left(x,t\right)\right]-F_{P}[1+V_{m^{*}}\cos\left(k\;x\right)]\left[m\left(x,t\right)\right],$$

where $F_{P}=k_{P}k_{I}\bar{I}/k_{T}$ is the average polymerization rate. Equation (7) can be normalized with the substitutions:

(8) $$\tau =t\;F_{P},\;\chi =x/\Lambda ,\;m=\frac{\left[m\right]}{\left[m_{0}\right]},\;R_{m}=\frac{D\;k^{2}}{F_{P}},$$

where $\left[m_{0}\right]$ is the formulated monomer concentration. This transforms Equation (5) into a unitless form:

(9) $$\frac{\partial }{\partial \tau }m\left(\chi ,\tau \right)=\frac{R_{m}}{{\left(2\pi \right)}^{2}}\frac{\partial^{2}}{\partial \chi^{2}}m\left(\chi ,\tau \right)-\left[1+V_{m^{*}}\cos\left(2\;\pi \chi \right)\right]\;m\left(\chi ,\tau \right),$$

which admits a standard harmonic solution $m\left(\chi ,\tau \right)=\sum_{i=0}^{4}m_{i}\left(\tau \right)\;\cos\left(2\;\pi \;i\;\chi \right)$, here expanded up to 4th order [12]. The resulting set of coupled first-order ordinary differential equations is:

(10) $$\frac{\partial }{\partial \tau }m_{0}\left(\tau \right)=-\;m_{0}\left(\tau \right)-\frac{V_{m^{*}}}{2}m_{1}\left(\tau \right)\;,\;\frac{\partial }{\partial \tau }m_{1}\left(\tau \right)=-\;V_{m^{*}}\;m_{0}\left(\tau \right)-\;\left(1+R_{m}\right)m_{1}\left(\tau \right)-\frac{V_{m^{*}}}{2}m_{2}\left(\tau \right),\;\frac{\partial }{\partial \tau }m_{1}\left(\tau \right)=-\;V_{m^{*}}\;m_{0}\left(\tau \right)-\;\left(1+R_{m}\right)m_{1}\left(\tau \right)-\frac{V_{m^{*}}}{2}m_{2}\left(\tau \right),\;\frac{\partial }{\partial \tau }m_{2}\left(\tau \right)=-\frac{V_{m^{*}}}{2}m_{1}\left(\tau \right)-\left(1+4\;R_{m}\right)m_{2}\left(\tau \right)-\frac{V_{m^{*}}}{2}m_{3}\left(\tau \right),\;\frac{\partial }{\partial \tau }m_{3}\left(\tau \right)=-\frac{V_{m^{*}}}{2}\;m_{2}\left(\tau \right)-\left(1+9\;R_{m}\right)m_{3}\left(\tau \right),\;\frac{\partial }{\partial \tau }m_{4}\left(\tau \right)=-\frac{V_{m^{*}}}{2}\;m_{3}\left(\tau \right).$$

These equations describe how the sinusoidal radical distribution of finite visibility given by $1+V_{m^{*}}\cos\left(2\;\pi \chi \right)$ drives the evolution of monomer concentration as a sum of grating harmonics. Consider the ideal recording case where diffusion is much faster than propagation such that $R_{m}\to \infty$. The equations show that all harmonics of the monomer higher than zero would remain identically zero, consistent with fast diffusion enforcing a spatially uniform monomer distribution. The average monomer concentration, $m_{0}\left(\tau \right)$, would then decay exponentially like exp(−τ) which is the ideal first order reaction with time constant $\tau =t\;F_{P}$ where $F_{P}$ is the average rate of polymerization.

Equation (10) is a sufficiently simple set of coupled first-order differential equations that it can be solved in closed form with the help of a symbolic mathematics package. With the monomer distribution m(χ,τ) known, the polymer distribution can then be found from the time integral of the last term in Equation (9):

(11) $$P\left(\chi ,\tau \right)=[1+V_{m^{*}}\cos\left(k\;\chi \right)]\overset{\tau }{\underset{0}{\int }}m\left(\chi ,\tau^{\prime }\right)\;d\tau^{\prime },$$

where P(χ,τ) is the polymer concentration normalized to $\left[m_{0}\right]$. P(χ,τ) represents the local concentration of monomer units that have reacted to become polymer. The left-hand side of Equation (11) is harmonically expanded as $P\left(\chi ,\tau \right)=\sum_{i=0}^{4}p_{i}\left(\tau \right)\;\cos\left(2\;\pi \;i\;\chi \right)$ and terms for each harmonic cos(2 π i χ) are set equal, generating integral equations for each polymer harmonic $p_{i}\left(\tau \right)$. These can also be solved in closed form with a symbolic math package. The resulting solutions to Equation (10) for the time evolution of the monomer harmonics, $m_{i}\left(\tau \right)$ and the solutions to Equation (11) for the time evolution of the polymer harmonics, $p_{i}\left(\tau \right)$, are a complete description of how the material responds to a sinusoidal intensity pattern. In the ideal recording case of perfect radical visibility, $V_{m^{*}}=1$, and very fast monomer diffusion, $R_{m}\to \infty$, the all polymer harmonics greater than two are zero. The average and first harmonic of the polymer become $p_{0}\left(\tau \right)=p_{1}\left(\tau \right)=1-\exp\left(-\tau \right)$ and, thus, P(χ,τ)=[1−exp(−τ)] [1+cos(2 π iχ)]. This is the ideal recording case where the polymer records a perfectly copy of the intensity.

With the monomer and polymer distributions known in space and time, the volume fraction of the matrix, $\phi_{M}$, can be found from the assumption of incompressibility, $\phi_{m}+\phi_{P}+\phi_{M}=1$. This completely specifies the composition of the trinary mixture. The composition and refractive indices of the constituents are then substituted into Equation (5), yielding a complicated but completely closed-form expression for material index as a function of exposure time. This lengthy equation depends on measured material parameters such as refractive indices, diffusion and reaction rates through the terms $R_{m}$ and $R_{m^{*}}$, hologram period and time. The first harmonic of this calculated index is then used to predict holographic efficiency as a function of reference beam angle via the beta value method [13]. The result is a completely closed-form prediction of hologram efficiency as a function of material parameters, writing conditions, and playback conditions.

The reason for developing this fully theoretical expression for holographic efficiency is that it can be compared to the numerical code suite which ideally implements the same physics but in a completely numerical, discrete manner. Specifically, in the limit of no scattering, the code suite will reproduce the intensity pattern, chemical reactions, distribution of species and refractive index to predict Bragg diffraction efficiency via the numerical techniques described previously. A comparison of the numerical to the theoretical solutions thus serves to validate the implementation and numerical approximations of the code suite.

**Figure 5:**
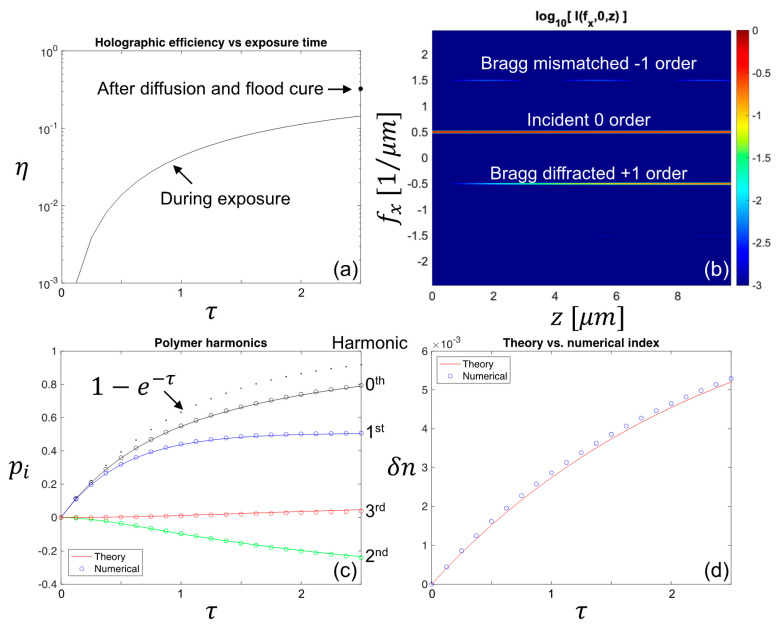
Comparison of 1 + 1D theoretical and 3 + 1D numerical predictions of grating growth versus time. The holographic diffraction efficiency shown in (**a**) exhibits the saturated response expected for an exposure time sufficient to consume all but $e^{-2.5}\approx 8\%$ of the monomer. Increased efficiency after dark diffusion and flood cure is consistent with slow monomer diffusion. A slice through the 3D numerical space shown in (**b**) is Fourier transformed in the transverse x coordinate to show the coupling of the incident light into the Bragg-matched +1 order and phase-mismatched coupling into the −1 order, illustrating that the numerical solution includes the complete physical description of the problem. In (**c**), a cross-section through the center of the numerical space is used to expand the index in a harmonic series at each time step, shown as circles. The closed-form theoretical prediction, rendered as lines, is in extremely good agreement. The dotted line shows the first-order kinetics expected for the zero and first harmonic in the case of perfect fidelity $\left(R_{m^{*}}\to 0\right)$ reaction limited $\left(R_{m}\to \infty \right)$ recording. The index contrast measured between the peak and null of the intensity is compared for the numerical (circles) and theoretical (line) cases in (**d**), again showing excellent agreement.

First, we use both the theory and numerical system to compare the evolution of the index grating itself. A 10 µm thick film of 20 wt% bisphenol A diacrylate in polyurethane matrix is exposed to two 405 nm Gaussian beams tilted to produce an unslanted 1 µm period transmission hologram. Radical diffusion is taken to be negligible $\left(R_{m^{*}}\to 0\right)$ but monomer diffusivity is finite and sufficiently slow $\left(R_{m}=0.2\right)$ to generate a significant second, but limited third and higher, harmonics. The normalized exposure time, τ=2.5, is sufficiently long to consume over 90% of the monomer. The solid lines in Figure 5c show the evolution of the fundamental harmonic recorded into the polymer, $p_{1}$, as well as the second harmonic, $p_{2}$, caused by the finite rate of monomer diffusion. The open circles show the fully numerical result, extracted by fitting a harmonic series to a slice through the 3D sampled grid. The excellent agreement between the closed form 1 + 1D and numerical 3 + 1D approaches validates that the numerical suite has faithfully reproduced the desired physics. Next, both the theoretical and numerically derived first harmonics of the polymer and monomer are combined through the Lorentz–Lorenz equation to predict the growth of the first harmonic of the refractive index, as shown in Figure 5d. The solid line is a completely closed form prediction of grating strength and the numerical suite accurately reproduces this prediction.

**Figure 6:**
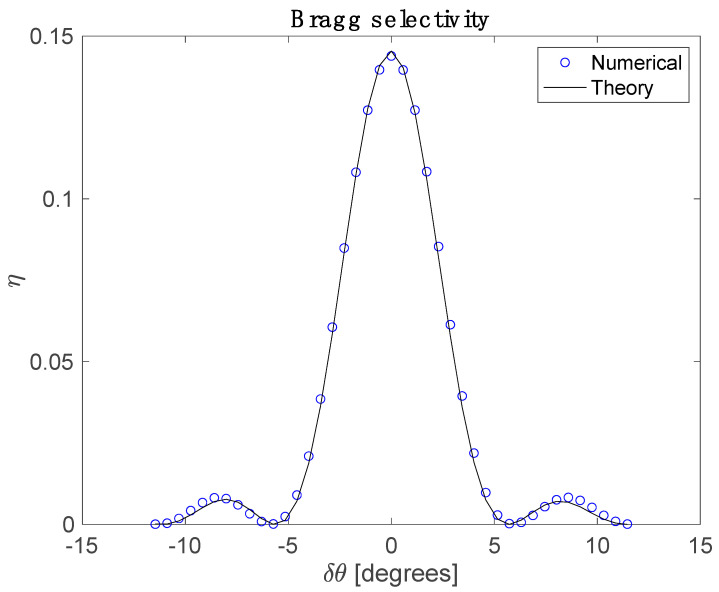
Solid line: Theoretical calculation of Bragg selectivity of a 10 µm thick sample of 20 wt% bisphenol A diacrylate in a solid polyurethane matrix illuminated with Gaussian beams at 405 nm to form a one µm period hologram with $1/e^{2}$ field diameter of 200 µm. The peak intensity is chosen to operate in the diffusion-limited regime where higher-order fringe harmonics are expected $\left(R_{m}=0.2\right)$. The exposure time is 2.5 exponential time constants of the mean polymerization rate, that is the monomer concentration is reduced to $e^{-2.5}$ of its initial value, averaged over a holographic fringe. Circles: The same calculation performed by the numerical code described in the Methods section. The reference probe beam has a $1/e^{2}$ intensity diameter of 40 µm. This reduced efficiency due to the overlap of the Gaussian hologram shape and the Gaussian read beam has been corrected as described in [14].

To further validate that the code correctly implements volume diffraction of off the numerically calculated index structure, a Bragg detuning curve found from both methods is compared. In the theoretical case, the calculated first harmonic of the refractive index is used in the beta value method [13] to predict the efficiency as a function of incident beam angle relative to the initial reference beam angle. In the numerical case, the three-dimensional space is illuminated with a tilted reference beam and the electric fields at the far side of the material are calculated via the FBPM method. The diffracted power is found by integrating the intensity in a range of spatial frequencies centered on the diffracted object beam. This is repeated for a series of reference angles. To compare the beta value method prediction of plane-wave diffraction off of an infinite grating to the numerical calculation of a Gaussian shaped hologram read with a Gaussian shaped reference beam, the theoretical efficiency is divided by $\left(1+2{\left(w_{0}^{read}/w_{0}^{write}\right)}^{2}\right)$ where $w_{0}$ is the 1/*e* radius of the electric field [14]. Figure 6 shows that the two approaches agree extremely well.

## 4. Results

The validated code is now applied to investigate how noise and signal develop in a holographic photopolymer containing scatter centers. The holographic photopolymer uses bisphenol A diacrylate (BPADA) as a writing monomer in a polyurethane matrix initiated with 2,4,6-trimethylbenzoyl diphenylphosphine oxide (TPO) at 405 nm. At 633 nm, the refractive indices of the monomer, polymer and matrix are 1.545, 1.5789, and 1.476 respectively. Dispersion of the indices is not included in this study. The shrinkage of the monomer in matrix upon full polymerization is found via the slanted grating technique [15] to be σ=0.14, consistent with typical vales of 22–24 mL/mol for complete shrinkage of acrylate monomers. Initiator concentration is sufficiently low that the samples may be considered optically thin and, thus, no absorption is included. Rather than use measured diffusivity, reaction rates, initiator concentration and intensity to calculate a specific value for the diffusion to reaction rate ratios for monomer and radicals, $R_{m}$ and $R_{m^{*}}$, we will take these to be free variables in order to explore their role in haze development. Note that these ratios are not fixed even for a specific material since they depend on hologram period and optical intensity, thus, they can be varied experimentally to exploit the trends shown next. Similarly, we take initial haze to be a free variable to understand its importance in haze development.

The writing beams are tilted, except where stated, to produce a grating period of one µm and have a 1/*e*^2^ intensity diameter of 200 µm. The readout beam used for nondestructive measurement of diffraction efficiency and haze is at 660 nm with a 40 µm 1/*e*^2^ intensity diameter, tilted at the appropriate Bragg matched angle or normally incident, respectively. Initial haze is generated by Rayleigh scatter centers with number density $10^{-3}/\mu m^{3}$ whose cross-section is adjusted to give the desired initial haze.

**Figure 7:**
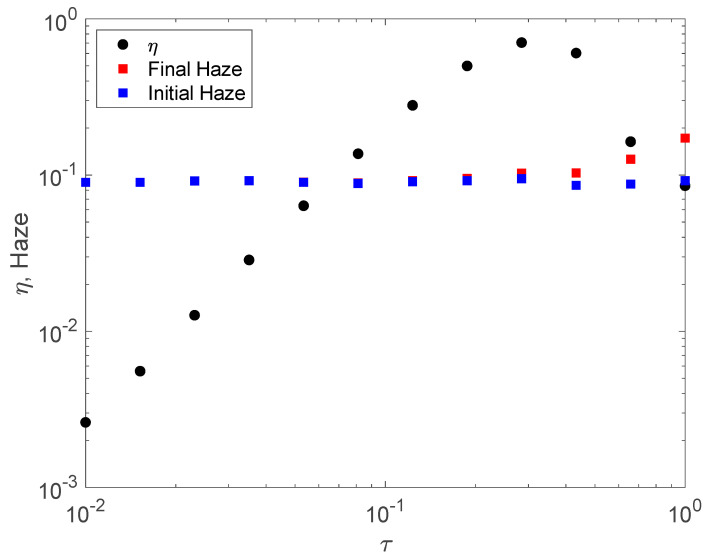
Typical grating and haze growth versus exposure time in the weak regime for 10 wt% BPADA operating in the reaction-limited regime ($R_{m}=400$). Haze is observed to only grow significantly above the initial value of approximately 0.1 when the hologram efficiency is saturated, in this case in the over-modulated regime.

Typical growth dynamics are shown in Figure 7. Even for this case with relatively high initial haze of nearly 0.1, haze is observed to grow much more slowly than diffraction efficiency. In this case, the hologram is overmodulated before significant haze growth occurs. This observation is consistent with the hypothesis that noise gratings are written by interference of writing beams and scattered light while the intended grating is written by the much stronger interference of reference and object beams. The first result of this numerical technique is therefore that significant haze growth arising from amplification of Bragg-matched noise gratings only occurs for holograms that are strongly saturated.

**Figure 8:**
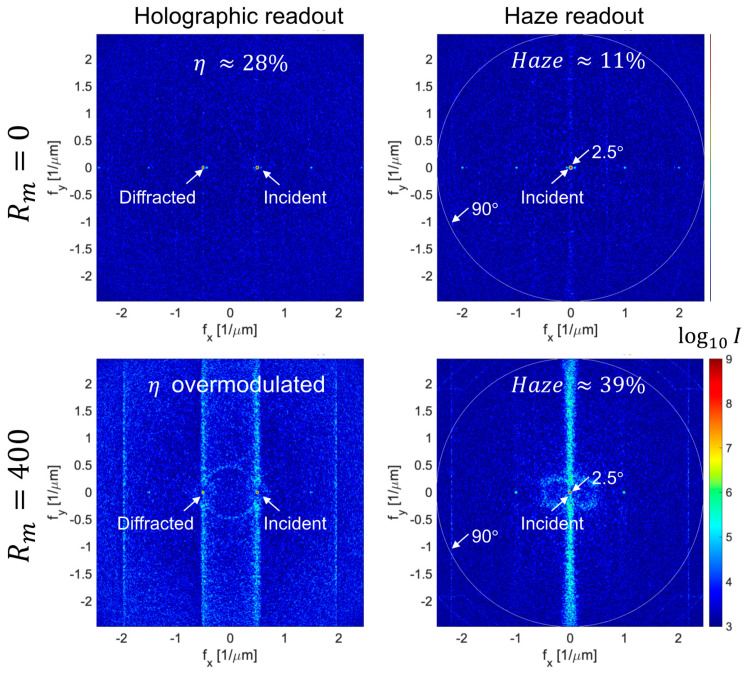
Typical strong recording results for holographic (**left**) and haze (**right**) readouts in the case of slow (**top**) and fast (**bottom**) diffusion rate across a period relative to average polymerization rate. Only the lower case exhibits the scattering arcs and lines characteristic of Bragg-degenerate noise gratings. Intensities are plotted versus transverse spatial frequency in the material, rendered with a logarithmic color map to visualize the large dynamic range. Monomer loading in these 100 μm thick films is reduced from the previous plot to 10 wt% and initial haze is set to ≈9%. Exposure time of τ=2.5 is sufficient to consume $1-e^{-\tau }\approx 92\%$ of the monomer.

Haze growth via amplification of Bragg-matched noise gratings is further supported by recording in diffusion and reaction-limited materials as shown in Figure 8. The distinct patterns visible in the spatial frequency spectrum of the scatter in the lower plots are consistent with Bragg degenerate noise gratings amplified during writing by fast diffusion. Conversely, when no diffusion occurs during writing, the haze growth is limited and shows no distinct patterns. This also serves to illustrate that haze growth in two-beam holography is distinctly different than self-written waveguides [3] in which scatter centers seed filaments whose scattering would be approximately isotropic in the transverse plane.

The top and bottom plots of Figure 8 reveal how recording dynamics impact grating and scatter development. In the top diffusion-limited regime of $R_{m}=0$, negligible haze has developed because the feedback process whereby noise gratings seed the growth of additional gratings has been suppressed. Simultaneously, the single strong hologram has been limited in its diffraction efficiency because monomer was unable to replenish that depleted in the bright regions of the fringe. Conversely in the bottom reaction-limited case of $R_{m}\to \infty$, the experimentally observed scatter patterns (see e.g., Figure 1) are observed. Diffraction efficiency is in the over-modulated regime since nearly all the monomer has been consumed due to rapid transport into the bright fringes. A multiplexed set of weaker holograms with appropriate scheduling would yield the same total usable index contrast in both cases, but the upper case would exhibit greater SNR.

**Figure 9:**
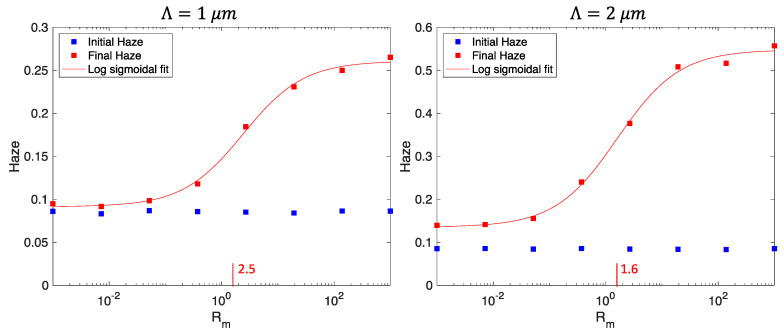
Haze development as a function of $R_{m}$, the ratio of diffusion rate across a period to average polymerization reaction rate for hologram period Λ=1 μm (**left**) and Λ=2 μm (**right**). The semi-logarithmic sigmoidal fit has an inflection point at $R_{m}=2.5$ (**left**) and $R_{m}=1.6$ (**right**), consistent with the hypothesis that haze development depends strongly on recording in the diffusion-limited ($R_{m}\ll 1)$ or reaction-limited $\left(R_{m}\gg 1\right)$ regime. The material is the same as in the previous figure.

The significant dependence of total haze upon the diffusion to reaction rate ratio shown in Figure 8 indicates $R_{m}$ has a critical role in haze development. Figure 9 plots the final haze versus $R_{m}$ under the same conditions as Figure 8. The plots show two distinct behaviors. When diffusion across a period is sufficiently slow that noise gratings cannot develop during the exposure, haze is limited to that which is recorded by the instantaneous index change. Conversely, when diffusion is sufficiently fast, noise gratings are self-reinforced during the exposure. The transition between these two regimes is seen to be approximately the boundary between diffusion-and reaction-limited systems at $R_{m}\approx 1$. Critically, this indicates that the relevant spatial scale for scatter is approximately equal to the period, Λ, consistent with the hypothesis that haze is not spatially random but is dominated by growth of Bragg degenerate noise gratings whose scale is similar to the grating period. This is contrast to the alternative hypothesis that the spatial scale of haze is similar to the particle size. Since scatter centers are typically much smaller than the period, noise dynamics controlled by diffusion would be much faster than signal dynamics in this case. The important implication for recording high SNR holograms is that both signal and noise obey similar dynamics such that diffusion-limited grating development will largely suppress haze development. The material and process variables that influence this condition are related by the single parameter $R_{m}$.

Since haze is seeded by scattering, an obvious strategy for reduction of haze is to reduce the initial scatter, e.g., via resin filtering and clean-room processing. Given that recorded haze is an amplified growth process, it is possible that reductions in initial haze could have super-linear impact on final haze. 

**Figure 10:**
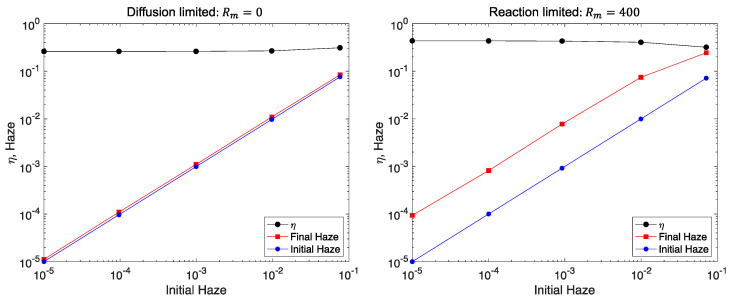
Final holographic efficiency (circles) and haze (squares) as a function of the initial haze. Haze is due to Rayleigh scattering for diffusion-limited (**left**) and reaction-limited (**right**) recording for the same material as Figure 8 and Figure 9. Final haze is seen to be a fixed multiple of initial haze if the signal and noise gratings are not competing for monomer, that is haze plus diffraction efficiency is much less than one. The dependence of haze growth on the relative rate of diffusion and propagation reaction is consistent with Figure 9.

Figure 10 shows how recorded diffraction efficiency and final haze scale with initial haze, controlled here via the cross section of Rayleigh scatter centers of the same number density of $10^{-3}/{\mu m}^{3}$. These results show that final haze is a fixed multiple of the initial haze. The ratio depends on the material and recording conditions. This result indicates that clean sample preparation is important, but has only linear impact on the final haze value.

**Figure 11:**
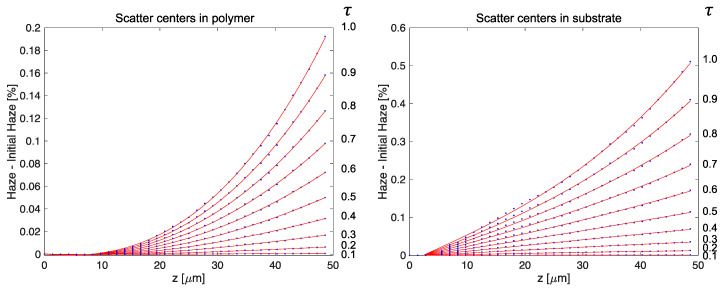
Haze growth above the initial scatter as a function of normalized exposure time τ and sample thickness, z. On the left, scattering centers are distributed randomly throughout the volume of the polymer, while on the right the same number of scattering sites are exclusively on the incident substrate. Data points produced by the numerical method are connected by cubic polynomial fits as visual guides. The transmission hologram has period Λ=1 μm and initial scatter is set to 0.5%. As in previous figures, the material is 10 wt% bisphenol A diacrylate in polyurethane.

Finally, we consider the dependence of recorded haze on sample thickness. It is commonly observed experimentally that haze grows rapidly with sample thickness and this is confirmed in Figure 11 where the initial haze has been subtracted to highlight the dynamics of haze growth caused by the medium. The left and right plots illustrate how uniformly distributed scatter (left) produces less haze than the same scatter concentrated on the substrate through which the light enters (right). Conversely, the distributed scatter (left) exhibits greater nonlinearity versus thickness, consistent with noise gratings being written by scattered intensity which grows linearly in depth (left) but is initially constant in depth (right). While we have fit this data to cubic polynomials to guide the eye, the thickness dependence of the haze does not appear to have any simple scaling relationship. This may be due to the complexity of the writing intensity pattern for haze which is generated by scatter from the static and dynamic haze interfering with the reference and object beams whose intensity varies in depth as they couple. Once significant monomer has been consumed (τ>0.5), the haze and the hologram also compete for monomer, slowing haze growth. This highlights the fact that complete understanding of haze dynamics requires a theoretical treatment. The numerical results and approach presented here may provide input to such an approach.

## 5. Conclusions

The results show that the development of both intended holographic signal and unwanted noise gratings in holographic photopolymer can be studied by numerically solving the coupled physics of optical diffraction, reaction, diffusion and index formation. The technique is demonstrated to accurately reproduce a closed-form solution to the two-beam recording condition including the shape and amplitude of the index grating as well as the Bragg selectivity curve. Numerical studies of signal and haze development reveal the dynamics of haze formation as a function of experimental parameters. Integrated haze is found to depend linearly on initial scatter strength and super-linearly on sample thickness. Haze growth is shown to be largely suppressed for diffusion-limited recording. This condition can be achieved via a number of experimental routes including high intensity, short exposures. The required intensity to record in the diffusion-limited regime can be found from the unitless parameter $R_{m}$, originally defined in [12], which is inversely proportional to intensity. In summary, the scaling relationships revealed by these numerical studies should provide routes for materials and process design to maximize SNR in demanding applications.

## Appendix A. Forward Difference Solutions to Reaction Rate Equations

Direct forward differencing of the chemical rate equations is only accurate to first order in the time step. To increase the accuracy of time stepping the reaction equations, we solve the local rate equations (that is, without diffusion) and use the resulting solutions for evolution of species. In the case of no radical diffusion, $R_{m*}=0$, Equation (9) that describes the monomer dynamics is thus reduced to:

(A1) $$\frac{\partial }{\partial \tau }m\left(\vec{r},\tau \right)=-I\left(\vec{r},\tau \right)\;m\left(\vec{r},\tau \right),$$

where $I\left(\vec{r},\tau \right)$ is the normalized intensity given by ${\left|E\left(\vec{r},\tau \right)\right|}^{2}$. The solution at an advanced time τ+δτ given a known monomer distribution at time τ is:

(A2) $$m\left(\vec{r},\tau +\delta \tau \right)=m\left(\vec{r},\tau \right)\exp\left(-I\left(\vec{r},\tau \right)\;\delta \tau \right).$$

Note that a series expansion of Equation (A2) agrees with the simple forward finite difference solution of Equation (A1) up to the first two terms.

For finite radical diffusion, $R_{m*}\neq 0$, the local update to radical concentration ignoring diffusion from Equation (1) becomes:

(A3) $$\frac{d}{d\tau }m^{*}\left(\vec{r},\tau \right)=\frac{R_{m}}{R_{m*}}\left(I\left(\vec{r},\tau \right)-m^{*}\left(\vec{r},\tau \right)\right),$$

where $m^{*}\left(\vec{r},\tau \right)$ is radical concentration $\left[m^{*}\left(\vec{r},t\right)\right]$ normalized to the steady state radical concentration found in Equation (2), $m^{*}\equiv \;[m^{*}]/\left(k_{I}\;\bar{I}/k_{T}\right)$. The ratio of diffusion to reaction rates $R_{m}/R_{m^{*}}$ in this equation arises because of the choice of propagation rate to normalize time in Equation (A1). The solution at an advanced time τ+δτ given a known radical distribution at time τ is:

(A4) $$m^{*}\left(\vec{r},\tau +\delta \tau \right)=\left(m^{*}\left(\vec{r},\tau \right)-I\left(\vec{r},\tau \right)\right)\exp\left(-\frac{R_{m}}{R_{m*}}\delta \tau \right)+I\left(\vec{r},\tau \right).$$

This radical distribution then reacts with monomer as in Equation (A2) via:

(A5) $$m\left(\vec{r},\tau +\delta \tau \right)=m\left(\vec{r},\tau \right)\exp\left(-m^{*}\left(\vec{r},\tau \right)\;\delta \tau \right),$$

replacing Equation (A2). After this reaction step is calculated, monomer and (if $R_{m*}\neq 0$) radicals are allowed to diffuse for one time step via the Fick’s law transfer function described earlier.
